# Electrocochleography and cognition are important predictors of speech perception outcomes in noise for cochlear implant recipients

**DOI:** 10.1038/s41598-022-07175-7

**Published:** 2022-02-23

**Authors:** Amit Walia, Matthew A. Shew, Dorina Kallogjeri, Cameron C. Wick, Nedim Durakovic, Shannon M. Lefler, Amanda J. Ortmann, Jacques A. Herzog, Craig A. Buchman

**Affiliations:** Department of Otolaryngology—Head and Neck Surgery, Washington University School of Medicine in St. Louis, 660 S. Euclid Ave, Campus Box 8115, St. Louis, MO 63110 USA

**Keywords:** Outcomes research, Sensory systems, Risk factors, Translational research

## Abstract

Although significant progress has been made in understanding outcomes following cochlear implantation, predicting performance remains a challenge. Duration of hearing loss, age at implantation, and electrode positioning within the cochlea together explain ~ 25% of the variability in speech-perception scores in quiet using the cochlear implant (CI). Electrocochleography (ECochG) responses, prior to implantation, account for 47% of the variance in the same speech-perception measures. No study to date has explored CI performance in *noise*, a more realistic measure of natural listening. This study aimed to (1) validate ECochG total response (ECochG-TR) as a predictor of performance in quiet and (2) evaluate whether ECochG-TR explained variability in noise performance. Thirty-five adult CI recipients were enrolled with outcomes assessed at 3-months post-implantation. The results confirm previous studies showing a strong correlation of ECochG-TR with speech-perception in quiet (*r* = 0.77). ECochG-TR independently explained 34% of the variability in noise performance. Multivariate modeling using ECochG-TR and Montreal Cognitive Assessment (MoCA) scores explained 60% of the variability in speech-perception in noise. Thus, ECochG-TR, a measure of the cochlear substrate prior to implantation, is *necessary but not sufficient* for explaining performance in noise. Rather, a cognitive measure is also needed to improve prediction of noise performance.

## Introduction

Cochlear implantation has become an effective treatment option over the past 3 decades for individuals with severe-to-profound hearing loss. Although cochlear implantation is now more common, performance as measured by speech-perception outcomes is highly variable and remains largely unexplained^1–5^. The wide variability in cochlear implant (CI) performance postoperatively makes it challenging to effectively counsel patients on realistic expectations. Recognizing factors that affect CI performance at a preoperative candidacy level may have significant implications on post-CI aural rehabilitation, device design and fitting, and surgical technique.

Early work in the 1990s suggested that duration of deafness and age at implantation negatively affected speech-perception outcomes after implantation^2,6^. When a similar study was repeated in 2013, these two variables accounted for less than ~ 20% of the variance in CI performance^4,5^. This was thought to be due to a shift in the patient population over the last 2 decades, where patients now are undergoing implantation with shorter durations of deafness.

Another factor more recently shown to be correlated with CI performance is electrode positioning within the cochlea. Studies have used either postoperative x-rays or computed tomography (CT) scans to determine insertion depth, number of electrodes in scala tympani, and distance from the modiolus. With the development of postoperative 3-D CT reconstructions, the addition of these imaging findings, evaluating the position of the electrode within the cochlea, together with duration of hearing loss, and age at implantation have still only been able to account for ~ 25% of the variance in CI performance^7^. Generally speaking, the weak correlations of the biographical and surgical factors suggest these variables are poor biomarkers of the underlying peripheral and central auditory substrate needed to support effective CI stimulation and thus, performance.

Recently, electrocochleography (ECochG) has been repurposed from a Meniere’s disease diagnostic application to investigating its role in improving CI outcomes. The intraoperative ECochG recording has generally performed at the round window (RW), prior to array insertion, and measures acoustically evoked electrical potentials from various structures within the cochlea. Hair cell function can be extrapolated from the cochlear microphonic (CM) and summating potential (SP), where the outer hair cells generate the CM potential^8^ and the inner hair cells contribute more to the SP^9^. The neural function can be measured from the compound action potential (CAP), which is representative of synchronous action potential across numerous fibers in response to an acoustic stimulus. The auditory nerve neurophonic (ANN) is the phase-locked neural firing throughout the duration of a tone; this can only be visualized when alternating polarity tones are used and is a sinusoidal wave of twice the stimulus frequency^10^.

A summative single measure of residual cochlear function, ECochG total response (ECochG-TR), can be calculated from ECochG responses by summing the tonal stimuli of different frequencies across the speech spectrum. The ECochG-TR has been found to explain just under half of the variance (47%) of postoperative CI speech-perception performance in quiet testing measures^11–13^. Independently, the ECochG-TR can explain the variance in CI performance better than demographic, audiometric, and surgical factors. The addition of demographic and audiometric variables to ECochG-TR in a multivariate model does not improve the prediction of CI performance, suggesting that these variables are likely represented within the ECochG-TR^12^. A major limitation of prior work utilizing ECochG-TR is that it has not been replicated outside of the original study institution. Also, this prior work has primarily focused on predicting performance in quiet.

Most CI users achieve excellent speech perception in quiet, but at least half continue to have poor performance in noisy settings^14–17^. This can be particularly challenging when there is no spatial separation between the stimulus and background noise. As listening in background noise is representative of most real-life hearing situations, poor performance in background noise can lead to significant communication problems; and in prelingual patients, this can lead to negative cognitive and linguistic development^17–19^. Preoperative cognition and linguistic skills and the signal processing of the CI device itself have been suggested to contribute to CI performance in background noise^20,21^. To our knowledge, no study has explored the prediction of CI performance in noise using a central, cognitive measure and ECochG-TR.

The objective of the present study was to identify sources of variability in CI outcomes by evaluating recently implanted, adult CI recipients (n = 35) from a single center. This prospective study was designed (1) to validate the work by Fitzpatrick et al.^11^ using intracochlear ECochG-TR to predict CI performance in quiet and (2) to explore ECochG-TR and other audiologic and demographic variables as predictors of CI performance in background noise. Based on previous studies, we expected that ECochG-TR would be a strong, independent predictor of performance in quiet. We hypothesized that ECochG-TR and a measure of global cognitive function, based on its relationship to the central auditory system, would contribute significantly to the variability of CI performance in noise.

## Results

### Participant demographics and characteristics

**Figure 1:**
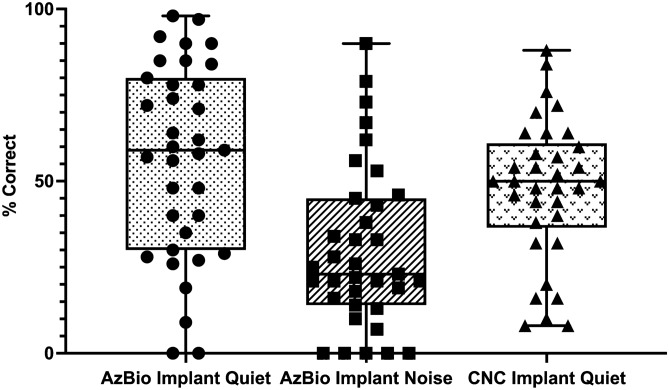
Box plots of performance measures showing all data points of 35 patients at 3 months across AzBio in Quiet, AzBio in Background Noise, and CNC in Quiet. All measurements were made with the CI-only condition. There was substantial variability across all speech recognition measures among CI recipients.

Of the 35 patients included within the study, 68.6% (24/35) were male with a mean age of 71.4 ± 16.4 years at the time of surgery. As in previous studies^22^, there was wide variability in CI performance both in quiet and noise testing measures post-implantation (Fig. 1). Poor performers were defined as those > 1 standard deviation from the mean AzBio or consonant-nucleus-consonant (CNC) scores (i.e., 28.1% and 26.9%, respectively). Further demographic information can be found in Table 1.

**Table 1:** Demographic information for all 35 patients who met inclusion criteria for the study and underwent cochlear implantation with 3-month follow-up for intracochlear electrocochleography recordings.

	Mean ± STD or N (%)
**Electrode array type**	
Perimodiolar	12 (34.3%)
Lateral Wall	23 (65.7%)
**Laterality**	
Right	24 (68.6%)
Left	11 (31.4%)
Low-frequency pure tone average (LFPTA; 125, 250, 500 Hz; dB HL)	79.8 ± 23.3
Pure tone average (PTA; 500, 1000, 2000, 4000 Hz)	99.9 ± 17.6
Montreal Cognitive Assessment (MoCA)	25.1 ± 4.9
**Hearing preserved at 3 months**	
Yes	14 (40.0%)
No	21 (60.0%)
Age (years)	71.4 ± 16.4
Duration of hearing loss (years)	24.8 ± 16.0
Duration of severe/profound hearing loss (years)	10.6 ± 14.6
**Etiology of hearing loss**	
Progressive	18 (51.4%)
Unknown	8 (22.9%)
Meniere’ disease	1 (2.9%)
Sudden sensorineural hearing loss	3 (8.6%)
Meningitis	1 (2.9%)
Otosclerosis	1 (2.9%)
Congenital	3 (8.6%)
Length of hearing aid use (years)	16.4 ± 15.5
**Gender**	
Female	11 (31.4%)
Male	24 (68.6%)
**Asymmetric hearing loss**	
No	34 (97.1%)
Yes	1 (2.9%)
**Electroacoustic stimulation use**	
No	26 (74.3%)
Yes	9 (25.7%)
**Prior contralateral cochlear implant**	
No	28 (80.0%)
Yes	7 (20.0%)
**History of ipsilateral otologic surgery**	
No	33 (94.3%)
Yes	2 (5.7%)

### Audiogram and ECochG response

**Figure 2:**
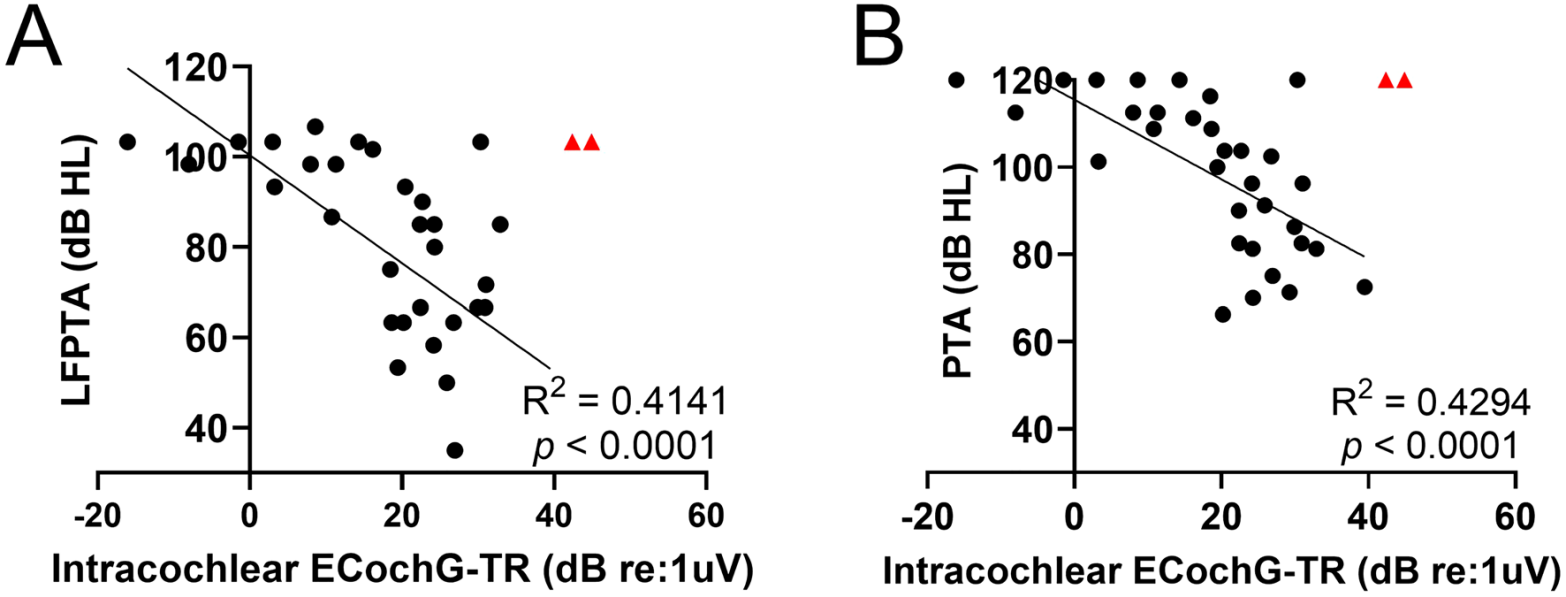
Univariate regression shows a moderate linear correlation between audiogram and intracochlear electrocochleography total response (ECochG-TR). Behavioral audiogram thresholds are likely a cumulative measure of both peripheral (i.e., auditory nerve and hair cells) and central auditory functioning; in contrast, intracochlear ECochG can provide direct and separate measures of acoustically evoked responses from the auditory nerve and hair cells. (**A**) Low-frequency pure tone average (LFPTA; 125, 250, 500 Hz) as a function of intracochlear ECochG-TR, and (**B**) Pure tone average (PTA; 500, 1000, 2000, 4000 Hz) as a function of intracochlear ECochG-TR. Red triangles denote outliers that were removed for univariate linear regression as these patients had auditory neuropathy spectrum disorder with large cochlear microphonic, contributing significantly to the ECochG-TR, and no neural response.

The mean pure tone average (PTA; 500, 1000, 2000, 4000 Hz) was 99.9 ± 17.6 dB HL and mean low-frequency pure tone average (LFPTA; 250, 500, 1000 Hz) was 79.8 ± 23.2 dB HL. ECochG-TR had a moderate-to-strong correlation with LFPTA (*r* = 0.64, *p* < 0.0001), and the correlation with ECochG-TR and PTA was similar with *r* = 0.66 (*p* < 0.0001; Fig. 2). Notably, we were able to measure ECochG responses in patients with LFPTA of 118.3 dB HL and PTA of 120 dB HL, which demonstrated the high sensitivity of ECochG responses and recording equipment. There were two patients excluded from the analysis as outliers, and upon further investigation (red triangles; Fig. 2) were found to have findings consistent with auditory neuropathy spectrum disorder (ANSD; Supplementary Fig. 1).

### Extra- and intracochlear ECochG recordings

**Figure 3:**
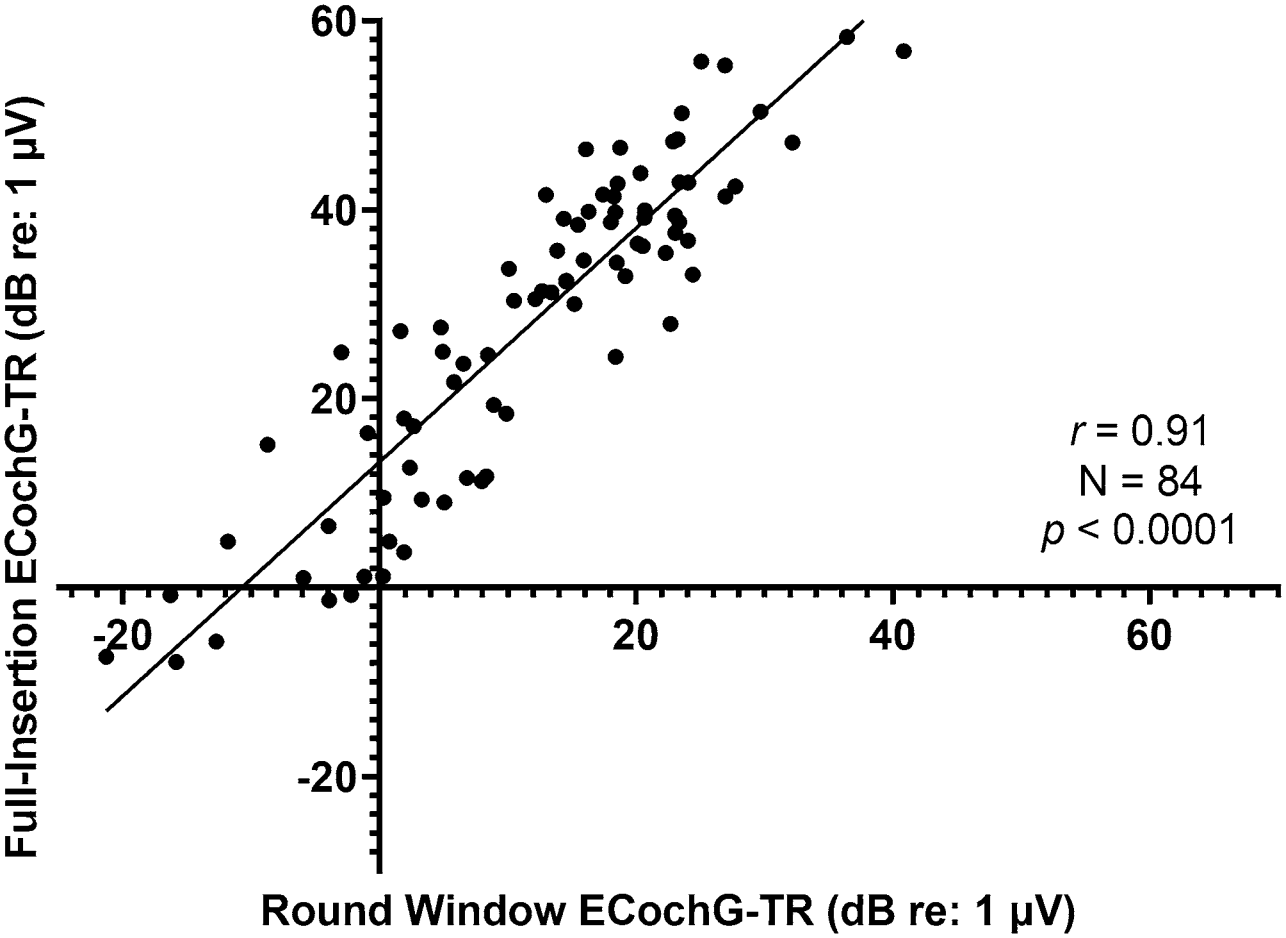
Simple linear regression shows a strong linear correlation for electrocochleography-total response (ECochG-TR) between round window and full insertion performed intraoperatively at the time of cochlear implantation.

ECochG-TR was measured intraoperatively at the RW and after full insertion. The mean ECochG-TR (measured in dB relative to 1 μV) was 13.8 ± 12.8 and 32.4 ± 16.9 for RW and after full insertion, respectively. There was a strong linear correlation between the ECochG-TR measured at the RW and after full insertion (N = 84, *r* = 0.91, *p* < 0.0001 Fig. 3). The amplitude of the full insertion ECochG-TR was ~ 8-times the amplitude of the RW ECochG-TR.

### ECochG and CI performance in quiet

**Figure 4:**
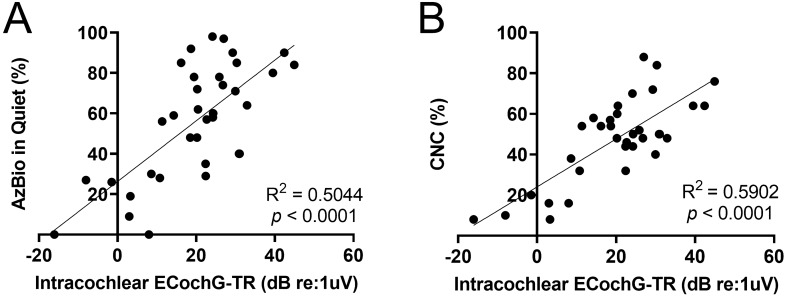
Univariate regression shows a moderate-to-strong linear correlation between performance in quiet and intracochlear electrocochleography total response (ECochG-TR). (**A**) Linear regression between ECochG-TR and AzBio in Quiet shows a moderate correlation, and (**B**) Linear regression between ECochG-TR and CNC shows a strong correlation.

ECochG responses were measured in the clinic at the patient’s 3-month postoperative visit to correlate with performance measures. The mean CNC word score was 47.6 ± 20.7% at 3 months. A linear regression model with ECochG-TR as the predictor indicated that ECochG-TR accounted for 59% of the variance in word scores as measured by CNC using the CI (Fig. 4). The mean score of AzBio in quiet was 56.3 ± 28.1% at 3 months. The ECochG-TR accounted for 50% of the variance in AzBio scores (*p* < 0.0001).

Multiple linear regression modeling was performed to determine whether the variability in performance in quiet, as measured by both AzBio and CNC, could be explained by other preoperative demographic or audiologic variables. Univariate regression was used to explore the association of AzBio and CNC with demographic and audiologic variables including duration of severe-to-profound hearing loss, electrode type, age, etiology of hearing loss, electroacoustic stimulation, bilateral CIs, audiogram, hearing aid use, and largest ECochG response at a non-apical electrode; none of these variables were significant predictors of speech perception scores. Variability of CI performance in quiet was poorly explained by duration of severe-to-profound hearing loss (*R*^2^ = 0.08, *p* = 0.04). A multivariable linear regression model with preoperative and postoperative PTA, electrode type, hybrid stimulation, duration of severe-to-profound hearing loss, and ECochG-TR did not perform better than ECochG-TR alone in explaining the variability in AzBio and CNC scores in quiet.

### Speech-perception performance in quiet and noise

The mean score (± SD) for the AzBio + 10 dB signal-to-noise ratio (SNR) in background speech babble was 27.0 ± 22.1%, ranging from 0 to 90%. As previously demonstrated^23^, there was a strong linear correlation between AzBio in Quiet and CNC (*r* = 0.87, *p* < 0.0001). There was a strong linear correlation (*r* = 0.75) between CNC (words measured in quiet) and AzBio + 10 dB SNR (Supplementary Fig. 2). A similar strong linear correlation was seen between AzBio in Quiet and AzBio + 10 dB SNR (Supplementary Fig. 2, *r* = 0.80, *p* < 0.0001). The analysis excludes two outliers as noted by red triangles in Supplementary Fig. 2A—one patient had the same performance in quiet as in noise which was suspicious for the audiologist not testing the patient with background babble and the second patient had a higher performance in noise than in quiet which may have been a documentation error.

### ECochG and CI performance in noise

**Figure 5:**
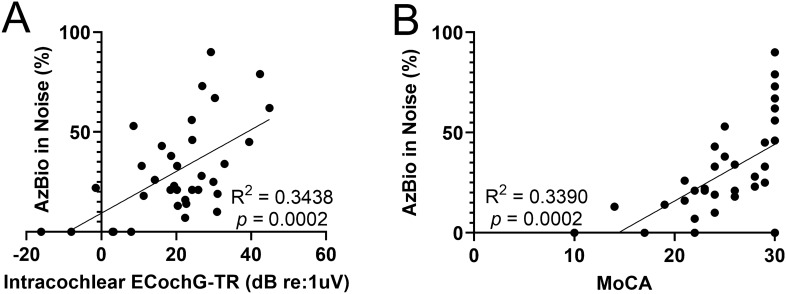
Independent univariate linear regression for predictors of performance in noise: (**A**) Electrocochleography total response (ECochG-TR) is a moderate predictor for performance in noise (AzBio + 10 dB SNR), (**B**) Montreal Cognitive Assessment score (MoCA) demonstrates a moderate linear correlation with performance in noise.

A linear regression between ECochG-TR and the dependent variable AzBio + 10 dB SNR was then performed to evaluate the predictive power of ECochG-TR on performance in noise. There was a moderate linear correlation between these two variables (*r* = 0.58, *p* = 0.0002; Fig. 5A). The slope of the regression line was 0.47; thus, for every 2 dB increase in ECochG-TR, there was ~ 1 percentage increase in AzBio + 10 dB SNR score.

### Cognition and speech-perception in noise

Model diagnostics were evaluated and multicollinearity between variables was assessed. The independent variables included ECochG-TR, Montreal Cognitive Assessment (MoCA) score, and duration of severe-to-profound hearing loss. The overall model predicted performance in noise (AzBio + 10 dB SNR) at 3 months after surgery (*F*(4,29) = 13.18, *p* < 0.0001), and accounted for 64.5% of the variance (*R*^2^). Table 2 summarizes the hierarchical multiple regression models explored. Through this approach, the proportion of the variation in CI performance in noise explained by the addition of our independent variables (MoCA, ECochG-TR, and duration of severe-to-profound hearing loss) was evaluated. Inclusion of MoCA into the model had the greatest impact upon prediction of AzBio + 10 dB SNR (*R*^2^ = 0.34, *p* = 0.0002; Fig. 5B). A significant interaction effect was observed between ECochG-TR and MoCA, and inclusion of this two-way interaction improved the model significantly (model 3, *R*^*2*^ = 0.60, *ΔF* = 2.07, *p* < 0.0001). Inclusion of other factors (i.e., duration of severe-to-profound hearing loss, type of electrode array, etiology of hearing loss, electroacoustic stimulation, bilateral CIs, audiogram, length of hearing aid use, largest ECochG response at non-apical electrode) improved the model little. For univariate regression, only age had a significant linear correlation with CI performance in noise (*r* = 0.42, *p* < 0.01); however, this was insignificant when added to MoCA for prediction of performance in noise.

**Table 2:** The hierarchical multiple regression analysis for predicting AZBio + 10 SNR scores with four different models (model 1: addition of electrocochleography total response (ECochG-TR), model 2: addition of Montreal Cognitive Assessment (MoCA) score, model 3: addition of two-way interaction between MoCA and ECochG-TR, model 4: addition of duration of severe/profound hearing loss (HL)).

	Model 1	Model 2	Model 3	Model 4
Variable	β	β	β	β
Constant	9.34	− 36.69*	7.18	12.39
ECochG-TR	1.046**	0.77*	− 2.46	− 2.7*
MoCA	–	2.06*	0.3	0.31
ECochG-TR*MoCA	–	–	0.12*	0.13**
Duration of severe/profound HL	–	–	–	− 0.36
*R*^2^	0.34	0.5	0.6	0.65
*ΔR*^2^	0.34	0.16	0.1	0.05
*ΔF*	17.29**	1.33	0.74	2.04

*ΔR*^2^ represents the variation in the dependent variable explained by the specific independent variable. ECochG-TR (model 1) improved the prediction of postoperative performance in noise as measured by AzBio + 10 dB signal-to-noise ratio considerably (*ΔR*^2^ = 0.34, *ΔF* = 17.29).

β = standardized regression coefficient; *p =  < 0.05; **p =  < 0.001.

## Discussion

The wide variability of post-implant word and sentence recognition abilities after CI has been a long-standing issue, particularly the inability to predict whether a patient will be a poor or excellent performer. Understanding factors that affect this variability in performance could allow audiologists and surgeons to counsel patients more accurately regarding realistic expectations after CI. Prior studies have revealed certain audiologic and demographic factors that affect CI outcomes such as duration of severe-to-profound hearing loss, age at implantation, cognition, and location of electrode within the cochlea^2,5,6,22^. Even with robust datasets using multivariate modeling, these variables have only been able to account for ~ 25% of the variability in CI performance in quiet.

The health of the implanted cochlea and auditory nerve has been postulated to be of importance for performance in CI recipients^24^. Specifically, ECochG-TR, a measure of residual cochlear-neural activation to auditory stimulation, has been shown to account for ~ 50% of the variability independently in CI-alone speech-perception performance in quiet in adults^11–13,25^. Both the CM and ANN contribute to the ECochG-TR; however, the ANN is often much smaller in amplitude than the CM. Thus, ECochG-TR is primarily a measure of hair cell activity including those hair cells that may be disconnected from the auditory nerve. This single measure serves as a potential biomarker for cochlear health and the underlying neural substrate that is available for electrical stimulation^13,26^.

A major limitation of the prior ECochG-TR studies is that the work has yet to be replicated outside of the initial study institution and the univariate regression has only been able to explain variance of performance in quiet. The current study was designed to validate the previous work predicting performance in quiet and perhaps more importantly, using the ECochG-TR measure with additional variables in an attempt to explain the more clinically meaningful measure of CI performance in noise. The primary difference between the previous work^11,13^ and the current study is the use of intracochlear ECochG-TR in contrast to the extracochlear ECochG-TR used in prior studies.

### Audiogram and ECochG Response

McClellan et al. explored the predictive power of ECochG-TR and found that duration of deafness, preoperative PTA and LFPTA on audiogram, and age at implantation were not able to explain a greater portion of the variance in CI performance in quiet when compared to ECochG-TR alone^12^. We first explored the relationship between unaided hearing detection thresholds and ECochG-TR (Fig. 2). There was a moderate linear correlation between ECochG-TR and LFPTA or PTA (*R*^2^ = 0.41 and 0.43, respectively). For a patient to achieve a certain threshold on an audiogram, both functional auditory nerve and hair cells are required. Thus, a partial or complete disconnection between auditory nerve and hair cells cannot be independently assessed on audiogram. The ECochG-TR serves as a proxy of cochlear health by measuring both hair cell and auditory nerve function^11,13^. The fact that ears with poor behavioral thresholds can have robust ECochG responses and do well with CIs leads us to hypothesize that cochlear synaptopathy^27,28^ may explain a portion of the variance of the audiogram in these patients.

### Extra- vs intracochlear ECochG recordings

Calloway et al. first showed the feasibility of intracochlear recordings and compared them to RW responses^29^. In general, the amplitude of the ECochG response increased ~ threefold as the recording electrode was inserted a few millimeters inside scala tympani. However, no direct statistical comparison between the ECochG measures from these locations was previously performed. Here, we show there is a strong linear correlation between the ECochG-TR measured at the RW and after full insertion (*r* = 0.91). Even though the general morphology of the waveforms across 250 Hz to 2 kHz (i.e., CM and ANN) was similar across the various extra- and intracochlear positions, there were fine structure components of the ECochG response that were more clearly present with the intracochlear recording. For example, the CAP was often only seen during the intracochlear recording as it is often significantly smaller than the ongoing ECochG response and primarily visualized at higher frequencies as the rise time for lower frequencies is limited by the duration of the cycle^10,11^. Since most CI recipients have profound hearing loss at high frequencies, a significant portion of the extracochlear recording was limited to ECochG responses from low-frequency acoustic stimuli.

### CI performance in quiet and ECochG response

The first study objective was to validate the prior work focused on predicting performance in quiet using ECochG-TR. This was done independently at our institution, in a new patient cohort using newly developed analytical code for ECochG responses. Previous studies have shown that ECochG-TR independently can explain 40–50% of CI performance in quiet as measured by CNC at 6 months^11–13^. Similarly, in the current study, ECochG-TR was positively correlated with both CNC and AzBio in Quiet at 3 months for the CI-only condition with *R*^2^ = 0.59 and 0.50, respectively. Thus, as identified by Fitzpatrick et al. and confirmed herein for the first time, ECochG-TR explains substantially more of the variance in performance than any of the classically used demographic or audiologic variables including duration of severe-to-profound hearing loss, age at implantation, and cognition. A major distinction between the prior work and the present study is that Fitzpatrick et al. recorded on the RW (an extracochlear site) while the present study responses were from an intracochlear electrode. Since we established a strong linear correlation between the RW and intracochlear ECochG-TRs, this corroborates the ability to use intracochlear ECochG-TR as a measure of cochlear health similar to previous studies using RW to predict CI performance^11–13,25^. Recently, Valenzuela et al. showed that the intracochlear ECochG-TR did not correlate with CI performance (both in noise and quiet) using the SlimJ electrode (Advanced Bionics, USA), which is in direct contrast to the findings in this study^30^. A major limitation of this prior work is the small sample size (N = 20), with at last 5 subjects having device failures; thus, any correlations between performance and ECochG-TR are difficult to assess as the study was only adequately powered to assess feasibility of intracochlear ECochG recordings.

In our study, duration of severe-to-profound hearing loss was weakly correlated with AzBio in Quiet (11.8% of variance) and CNC (8.0% of variance). Age was also weakly correlated with AzBio in Quiet (16.9% of variance) and CNC (6.8% of variance). When age, duration of severe-to-profound hearing loss, electrode array type, and hybrid stimulation were added to ECochG-TR in a multivariate linear regression model to predict AzBio in Quiet and/or CNC, there was no significant improvement in the model’s predictive power. According to Occam’s razor, where the simplest explanation should be used to account for the existence of a phenomenon, ECochG-TR independently, without the addition of other preoperative demographic and audiologic variables, is the *most* important predictor for CI performance in quiet.

### Performance in quiet is highly correlated with performance in background noise

After validating the work on ECochG-TR using intracochlear recordings to predict CI performance in quiet, various predictors of CI performance in noise were investigated. The initial finding was that performance in noise was highly correlated with performance in quiet (Supplementary Fig. 2). Unlike ECochG-TR or other preoperative audiologic and demographic factors, post-CI performance in quiet cannot be measured preoperatively to predict performance in noise. Furthermore, not all patients that obtained high scores on AzBio in Quiet performed well in background noise suggesting that other variables, namely central auditory processing mechanisms such as squelch, are likely to be in play to excel at this skill. Apart from one outlier, there were no patients who performed poorly in quiet that performed well in background noise. However, there were patients who performed more poorly in noise than expected based on their good performance in quiet. Even though these findings may seem simplistic, it was imperative to establish that good performance in quiet is *necessary but not sufficient* for good performance in background noise.

### Cognition, ECochG response, and performance in background noise

The results of this study indicate that the intracochlear ECochG-TR contributes to CI performance in background noise. CI recipients with larger values for ECochG-TR generally performed better than those with small values (*R*^2^ = 0.34). However, not all patients with large ECochG-TRs performed well in background noise (Fig. 5). This was in contrast to the results noted for performance in quiet as all CI recipients with large values for ECochG-TR performed well in quiet (Fig. 4).

Recently, the role of cognition on CI performance has been briefly explored as a theoretical explanation for the difficulty in explaining the variability of CI performance in noise^22,31^. To our knowledge, previous studies have not identified any objective measures to explain the wide variability for performance in noise. Performance in noise is certainly a more realistic assessment of a CI recipient’s ability to function in the real world. Hearing well in noise is especially challenging when the target stimulus has no spatial separation from background noise. To simulate this condition in this study, hearing in noise was measured with the CI-only condition using AzBio + 10 dB SNR and the signal was at the same site (0-degree azimuth); thus, the primary mechanism measured was central squelch^32^. Two theories exist that complement each other to explain the poor performance in background noise for most CI recipients: (1) “bottom-up” processing, (2) “top-down” processing^20,21^. “Bottom-up” processing suggests that the CI device itself and its poor resolving capabilities in noise leads to a degraded signal. An intact cochlea in normal hearing individuals can perceive pitch, timing, and timbre, which all contribute to the resolution of speech from noise^33^. To date, there are limited spectral channels within the CI device making it unable to fully resolve the temporal fine structure required for understanding speech in background noise. Alternatively, “top-down” processing suggests that the brain level processing required to cope with the CI signal also plays a role in CI performance. Speech perception requires linguistic and cognitive resources to assist with the recognition of the message and working memory to store the speech signal for a long enough time period to improve resolution and store signals into long-term memory^33^. Those with poorer cognition or linguistic skills preoperatively are expected to have limited ability for resolving CI signals in background noise. The resolution of the CI device itself (i.e., “bottom-up” processing) was controlled for in this study as the manufacturer of the CI and number of electrodes was the same across all participants. We anticipated that individuals with poor MoCA scores would perform poorly in background noise.

Here, we show a moderate linear correlation between performance in background noise and MoCA (*R*^2^ = 0.34). Although both MoCA and ECochG-TR were moderate predictors of CI performance in noise, they were individually better than any previously described combination of preoperative demographic and audiologic variables (in quiet). Notably, there are CI recipients that had high MoCA scores who did not perform well in background noise. These were patients that had small values for ECochG-TR, or poor preoperative cochlear health measures. A high MoCA score could not compensate for a poor cochlear neural substrate to result in good performance in noise; likewise, a high ECochG-TR (good cochlear health) could not compensate for a poor MoCA score to result in good performance in noise. Whereas the MoCA provides insight on central cognitive function, the ECochG-TR is a measure of the peripheral (i.e., cochlear) auditory neural substrate. Using a multivariate model, MoCA, ECochG-TR, and the interaction of MoCA and ECochG-TR (product of ECochG-TR and MoCA) were able to explain 60% of the variance in performance in noise. Both a normal MoCA score (≥ 26) and a large value for ECochG-TR are required for excellent performance in background noise. Thus, a good ECochG-TR value is *necessary but not sufficient* for good performance in background noise. Recent work has shown that testing in noise disproportionately affects older patients^34^. Although there was a weak correlation between age and performance in noise (*R*^2^ = 0.18), the addition of age did not improve the model in the current study, and there was significant collinearity between age and MoCA score. We suspect that the impact of age on cognition and performance in noise is incorporated within the MoCA score.

### Limitations

While this study can account for a large portion of the variance in CI performance in noise, there are several notable limitations. Surgical variables from imaging (e.g., angular insertion depth, scalar location, modiolar proximity) were not included in the multivariate regression model. Recent work by Canfarotta et al. has shown that angular insertion depth together with ECochG-TR accounts for ~ 70% of the variance for performance in quiet^25^. Additionally, prior studies have consistently shown a strong correlation between superior speech-perception performance and scala tympani insertions^22,35–40^. Future studies will need to further explore the addition of the surgical variables described above to MoCA and ECochG-TR for predicting performance in noise. Long-term speech-perception performance is another variable that must be evaluated in the future. Improvements in speech-perception performance in quiet and noise testing environments with additional listening experience has been shown for some CI recipients^41^. However, the largest increase in performance from preoperative to postoperative intervals is at 3-months post-activation with a significantly smaller improvement from 3 to 6 months^42^. The expectation would be that the prediction models would perform similarly in the long-term.

Another consideration in the current dataset is that the inclusion of hybrid stimulation was not significant for predicting CI performance. Only 9 patients (25.7%) were using hybrid stimulation in this study. Prior studies have shown benefits of residual hearing and hybrid stimulation for performance in noise as these patients are known to exhibit greater spectral and temporal resolution^43–46^. Prospective investigations are currently underway to address the impact of these variables on CI performance. Last, all ECochG-TR measurements for correlation with performance in this study were performed postoperatively using an intracochlear electrode, unlike previous studies using RW ECochG-TR prior to array insertion. The previous studies have shown that postoperative changes (i.e., fibrosis and osteogenesis) occur predominantly at the lower basal turn and cochleostomy site^47–49^. Furthermore, Fontenot et al. showed that a significant portion of the ECochG-TR is derived from the response at the lower frequencies (located within the apical cochlea), which may explain the presence of robust intracochlear ECochG responses in this study^13^. Future studies will need to investigate whether and/or how the ECochG response changes postoperatively.

### Future directions

Ultimately, to optimize clinical utility, we foresee using ECochG recordings through a trans-tympanic approach on the promontory to make the information described herein available preoperatively. This will inform patient counseling and potentially modify surgical techniques, device design, and CI aural rehabilitation. Future studies will need to evaluate the feasibility of trans-tympanic recordings and whether these measurements correlate with CI performance.

Despite expected perioperative changes^47–50^ including fibrosis, potential interference between the electrode array and basilar membrane, and synaptopathy, there were robust ECochG responses at 3-months postoperatively that reflect the speech-perception performance at that timepoint. There may be a diagnostic role for ECochG for understanding poor performance postoperatively as this technology may provide insight on cochlear health. Once the expected CI performance is established, modifiable variables (e.g., electrode positioning, post-aural auditory rehabilitation, electrode programming) may be addressed to enhance performance on a per patient basis. Investigations are currently underway for characterizing postoperative ECochG responses for patients who perform poorly on speech-performance testing.

## Conclusions

As previously reported and validated within this study, ECochG-TR explains a large portion of the variance (59%) in speech perception outcomes in quiet among adult CI recipients. CI performance in background noise, a more realistic measure of natural listening, is largely understudied particularly in the context of ECochG. Performance in quiet was strongly correlated with performance in background noise, suggesting that good performance in quiet is *necessary but not sufficient* for good performance in noise. Performance in noise showed a moderate linear correlation with ECochG-TR (34%) and MoCA (34%), independently. A model including MoCA, ECochG-TR, and duration of severe-to-profound deafness accounted for 65% of the variance in postoperative AzBio + 10 dB SNR scores in the CI-alone condition. These findings highlight the relatively large role that both cognition and the auditory periphery, as measured by ECochG-TR, have in speech-perception performance in background noise among adult CI recipients.

## Methods

### Participants

This prospective, continuous enrollment study was open to all adult patients undergoing CI or presenting to the clinic for their 3-month postoperative visit. Study approval was obtained through the Institutional Review Board of Washington University in St. Louis and all experiments were performed in accordance with relevant guidelines and regulations. All participants provided verbal and written informed consent prior to participation. Patients undergoing revision CI or with malformed cochlear anatomy were excluded. Furthermore, patients without a patent external auditory canal were excluded as the acoustic stimulus is initially delivered via air conduction. Demographic information including sex, age at implantation, laterality, duration of hearing loss, duration of severe-to-profound hearing loss, etiology of hearing loss, length of hearing aid use, use of hybrid stimulation, history of otologic surgery, presence of bilateral sequential CIs, and asymmetry of hearing loss were collected (Table 1). Asymmetric hearing loss was defined as severe-to-profound hearing loss in the affected ear with normal-to-moderate hearing loss in the contralateral ear.

All patients were implanted with either a lateral wall or precurved array. In this population, there were 2 precurved arrays and 1 straight array, all with 22 electrode contacts and made by Cochlear Limited (Sydney, Australia). The precurved arrays were: (1) slim perimodiolar electrode (CI632) and (2) perimodiolar electrode (CI612). The lateral wall electrode was a Slim 20 lateral wall array (CI624). All patients used the same N7 processor.

### Audiometric and cognitive measures

As part of the standard clinical protocol at the study institution, all adult participants underwent a comprehensive audiometric evaluation by a licensed audiologist using a modified Hughson-Westlake procedure prior to surgery. Speech recognition ability was evaluated using the Northwestern University Auditory Test No. 6 (NU-6), a monosyllabic word test with a consonant-nucleus-consonant construction, presented at suprathreshold levels. Preoperative audiometric evaluation included audiometric thresholds from 125 to 8000 Hz; the pure tone average (PTA; 500, 1000, 2000, 4000 Hz) and low-frequency pure tone average (LFPTA; 125, 250, 500 Hz) were also calculated. Speech perception testing was presented at 60 dBA in the CI-alone condition in the soundfield (0-degree azimuth) at candidacy and at 3 months of listening experience using the CNC word test, AzBio in Quiet, and AzBio + 10 dB SNR as previously described^51,52^. From our experience using the slim perimodiolar electrode, the largest improvement in speech-perception performance for both quiet and noise testing measures is at 3-months post-activation, with smaller improvements at 6- and 12-months^53^. Furthermore, similar to residual hearing, it is unknown whether the ECochG response is maintained in the long-term after implantation. Thus, the 3-month time was selected to measure speech-performance outcomes in this study. Of note, in CI users with stable acoustic behavioral hearing postoperatively, there is also stability in ECochG responses^54^. Cognition was assessed by the Montreal Cognitive Assessment (MoCA) score, a cognitive screening measure^55^.

### Postoperative intracochlear ECochG recordings

**Figure 6:**
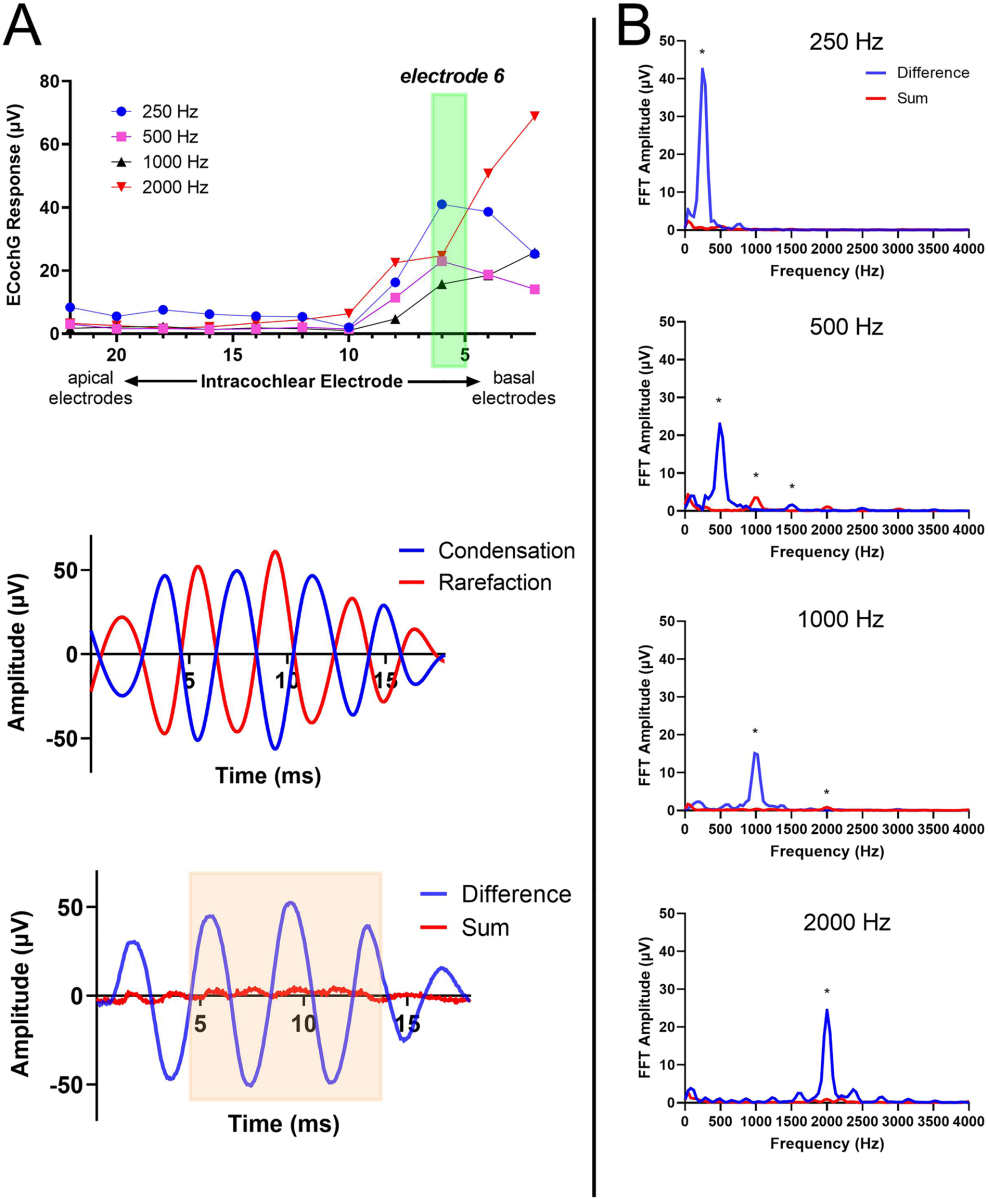
Sample ECochG recording and calculation of the electrocochleography total response (ECochG-TR). The electrode sweep is first collected across 4 frequencies using tone bursts—250, 500, 1000, and 2000 Hz (**A**, top panel). A representative response to 250 Hz tone burst is shown in (**A**, middle and lower panels). The stimulus alternated in phase, and both the condensation and rarefaction phases are shown (**A**, middle panel). The difference and sum waveforms are calculated from the condensation and rarefaction waveforms (**A**, lower panel). A window for the ongoing steady state response is selected from the difference and sum waveforms as shown in orange (4 to 14 ms) to compute the fast Fourier transformations (**A**, lower panel). (**B**) Magnitude spectra for each stimulus frequency with significant responses (250 to 2000 Hz). For each stimulus frequency, the magnitudes of the first three harmonic peaks were compared to the noise floor. Those that were significantly above the noise floor (asterisks, see text for test of significance) were added to produce the ECochG-TR.

ECochG potentials were measured from the electrode array itself using the Cochlear Research Platform Ver 1.1. An ER3-14A insert earphone (Etymotic, Elk Grove Village, IL) was placed into the external auditory canal. An electrode sweep was initially performed across all even electrodes, which resulted in recordings from a total of 11 electrodes. Tone burst (0.25, 0.5, 1, 2 kHz) stimuli were delivered, alternating in condensation and rarefaction starting phases, with 30 repetitions per phase. The rise and fall times were 1 ms and shaped by a Blackman window. For all frequencies, the recording epoch was 18 ms, starting 1 ms before stimulus onset, with a sampling rate of 20 kHz. Stimuli were presented at 104-, 98-, 97-, and 102-dB SPL for 250, 500, 1000, and 2000 Hz, respectively; these values were selected based on the maximum output of the speaker. A window isolating the ongoing portion of the response waveform was selected for a fast Fourier transformation (FFT) to evaluate the spectral characteristics of the response; this was performed within the Cochlear Research Platform Ver 1.1 for purposes of identifying the largest response on the electrode sweep. For all cases, the largest response was at one particular electrode across a majority of the frequencies that were measured (250, 500, 1000, 2000 Hz). The electrode with the largest response was then selected for a full frequency sweep measurement, with 100 repetitions per phase. The rest of the parameters were identical to the electrode sweep. If there were 2–3 electrodes with large responses across different frequencies, a frequency sweep was performed at those electrodes as well. A sample recording for electrode sweep, frequency sweep, and FFT are shown in Fig. 6. Prior work has shown that the ECochG response in CI recipients, largely the CM, does not disappear postoperatively, even when residual hearing is lost; however, there is a reduction in response compared to those measured before residual hearing is lost^50^.

For 71.4% of patients (25 / 35), the largest overall ECochG response was at the most apical electrode as identified on an electrode sweep. The apical electrode was then selected for a frequency sweep to calculate the ECochG-TR. For the other 10 patients, a non-apical electrode was selected for the frequency sweep based on the maximum ECochG response on the electrode sweep.

### Full-insertion vs round window ECochG recordings

RW recordings were performed intraoperatively in the setting of cochlear implantation. Similar to the postoperative recording, an earphone insert was placed in the external auditory canal prior to surgical site sterile preparation. A standard surgical approach was used for all CIs. Once there was adequate exposure of the RW via the facial recess, the CI was seated under the temporalis muscle. The telemetry coil was then placed over the skin in alignment with the CI antennae via a sterile ultrasound drape. The ground electrode was then placed on the RW, and a frequency sweep (250, 500, 1000, 2000 Hz) was performed using the ground electrode (ECE1) referenced to case ground (ECE2). After the recording, the ground electrode was placed under the temporalis muscle and the electrode array was inserted through the RW opening according to manufacturer’s specifications. The full-insertion ECochG-TR was performed after array insertion using the electrode with the largest response from the electrode sweep as discussed for the postoperative intracochlear ECochG recording above.

### ECochG signal analysis

ECochG responses were processed offline and stored as condensation and rarefaction phases. The ongoing portion of the response waveform was selected for FFT for each of the responses using MATLAB R2020a (MathWorks Corp., Natlick, MA) with custom software procedures. A significant response was defined as one whose magnitude exceeded the noise floor by 3 standard deviations; this has been well-defined in prior studies^11–13^. The noise floor’s variance and mean were defined by 6 bins, 3 on each side of the peak, starting 9 bins from the peak. The ECochG total response (ECochG-TR), a sum of the gross responsiveness of the cochlea was then calculated from the frequency sweep across the 4 frequencies (250, 500, 1000, 2000 Hz). The ECochG-TR is defined as the sum of the magnitudes of the significant responses at the first, second, and third harmonics across all frequencies. The electrode sweep was only used to determine which recording electrode had the largest response; however, this was not used directly for ECochG-TR calculation. The actual calculation of the ECochG-TR was performed post hoc, so the surgeon was blinded to the actual overall responsiveness of the cochlea during these measurements.

### Statistical analysis

Normality was confirmed using the Shapiro–Wilk test. Thus, the Pearson correlation (*r*) was used to determine strength of the relationships between ECochG-TR, PTA, LFPTA, MoCA score, and speech perception outcomes to understand factors that were independently important for CI performance in quiet and noise. Simple and multiple linear regression analyses were used to assess how well each of the variables and the combination of variables could be used to predict performance in quiet (i.e., CNC and AzBio in Quiet) and/or noise (AzBio + 10 dB SNR) at 3 months. The independent explanatory variables selected for the multivariate regression had p-values < 0.05 in the simple regression between CI performance and the respective independent variables. A hierarchical model approach with each of the variables added on at a time was used to evaluate incremental role of audiologic performance and cognitive scores over ECochG-TR alone in prediction of speech perception scores. Analyses were performed with SPSS 27 for Windows (IBM Corp, Armonk, New York, USA). Alpha level for all statistical tests was set at 0.05 and were two-tailed.

## Supplementary Information

Supplementary Figures.

## Data Availability

The MATLAB functions used in this study are available upon request from the corresponding author.
