# Supplementary material for: Electrocochleography and cognition are important predictors of speech perception outcomes in noise for cochlear implant recipients
Source: Sci Rep. 2022 Feb 23;12:3083. doi: 10.1038/s41598-022-07175-7 (PMC8866505; doi:10.1038/s41598-022-07175-7)

**Supplementary Figure 1**: The two outlier cases (A and B) where the electrocochleography-total responses (ECochG-TRs) were large, but the pure tone average and low-frequency pure tone average were larger than expected on linear regression. A frequency sweep from 250 to 2000 Hz at ~100 dB SPL was performed. There was minimal harmonic distortion within the ongoing ECochG response, suggesting that most of the response was from the cochlear microphonic with minimal contribution from the neural phase-locking. There was also no compound action potential across the recordings. Taken together, these findings suggest a minimal neural response to the acoustic stimulus.


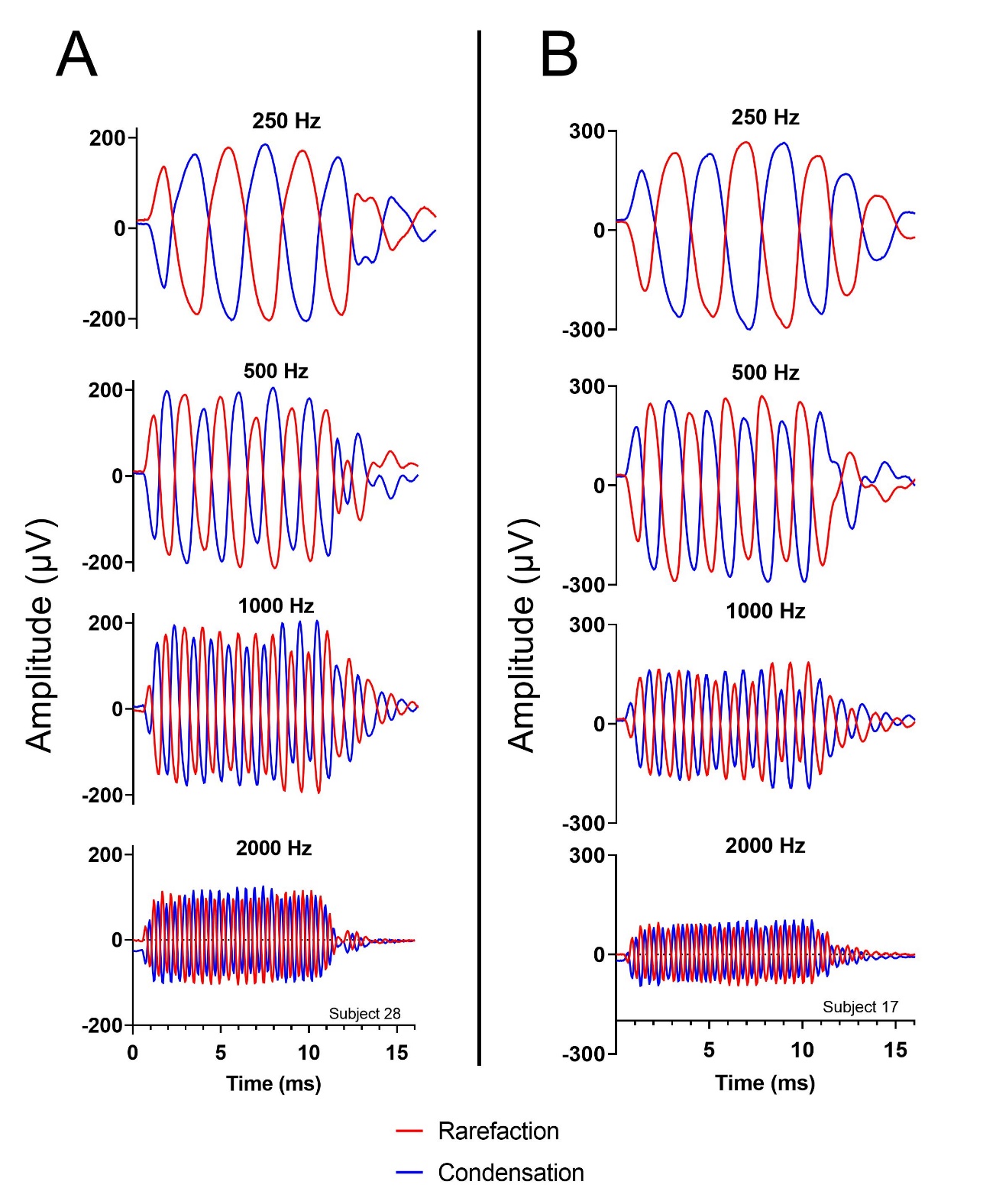


**Supplementary Figure 2**: Good performance in quiet (as measured by AzBio in Quiet) is necessary, but not sufficient for good performance in noise (as measured by AzBio in Noise). There is significant variability in performance in noise even in patients that perform well in quiet after CI in the CI-only condition. (A) There is a strong linear correlation between performance in quiet and performance in noise. Outliers are denoted in red – one subject obtained the same scores in quiet and in noise which was suspicious for potentially the noise environment not being added to the measure and the other subject had a higher score in noise than in quiet which may have been an error in documentation. (B) There is a strong linear correlation between performance in quiet (as measured by CNC) and performance in noise (as measured by AzBio +10 dB SNR).


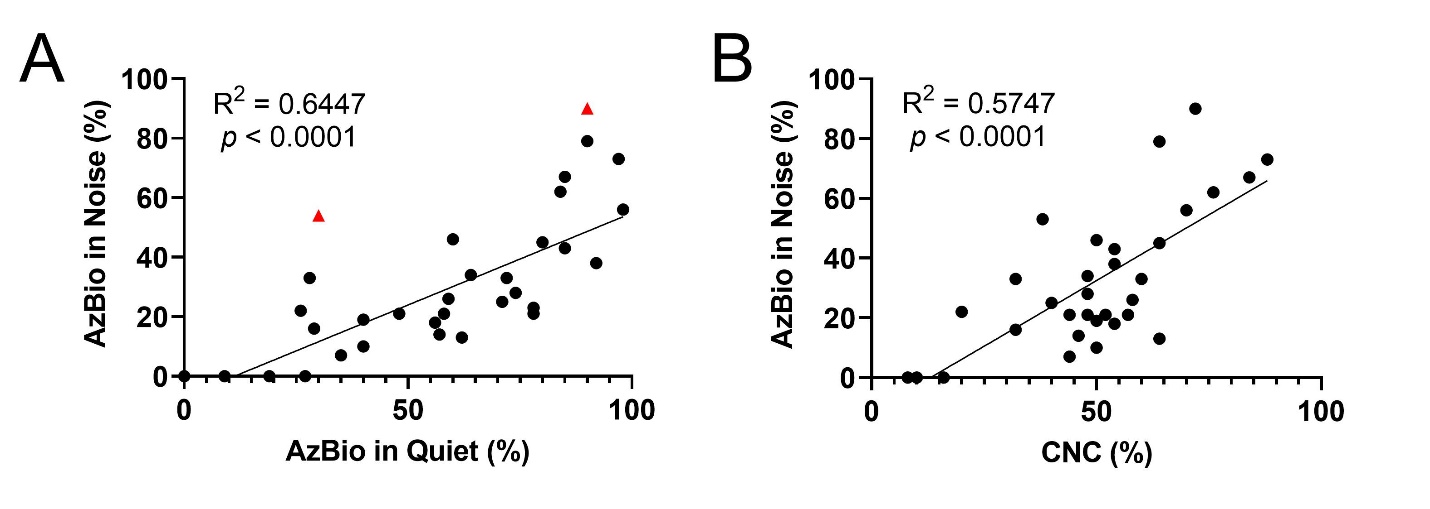

Supplement: Supplementary file 1 — Supplementary Figures. [file 41598_2022_7175_MOESM1_ESM.docx]
